# Influence of 5-HTTLPR polymorphism on postpartum depressive and posttraumatic symptoms

**DOI:** 10.1097/YPG.0000000000000299

**Published:** 2021-10-22

**Authors:** Marta Landoni, Sara Missaglia, Daniela Tavian, Chiara Ionio, Paola Di Blasio

**Affiliations:** a CRIdee, Psychology Department; b Psychology Department; c Laboratory of Cellular Biochemistry and Molecular Biology, CRIBENS, Università Cattolica del Sacro Cuore, Milan, Italy

**Keywords:** delivery, 5-HTTLPR, postpartum depression, posttraumatic stress disorder, pregnancy

## Abstract

Postpartum depression (PPD) is a multifactor disorder caused by psychological, social, and also biological factors. 5-HTTLPR polymorphism in the promoter region of serotonin transporter gene seems to influence PPD onset. In this study, we examined the effect of 5-HTTLPR polymorphism on prenatal and postnatal symptoms of depression and posttraumatic stress in women.

**Methods:**

A longitudinal design with three points – time 1 (32–40 weeks gestation); time 2 (2 or 3 weeks after birth), and time 3 (3 months after birth) – was made. A total of 141 women were recruited during childbirth preparation courses. At time 1, women completed the Beck Depression Inventory (BDI) and the Los Angeles Symptoms Checklist (LASC). At time 2, they fulfilled BDI and Edinburgh Postnatal Depression Scale (EDPS), LASC and the Perinatal Posttraumatic stress disorder (PTSD) Questionnaire (PPQ); midwives and nurses collected biological test tubes by blood sampling for the genetic analysis. At time 3, the women were reassessed for BDI, LASC, EDPS, and PPQs. Analysis of variance and moderation analysis were used to correlate genotype and psychological investigations.

**Results:**

Results showed that, compared with LL/LS genotypes, SS genotype moderated cognitive depressive symptoms onset at T2 and T3. Moreover, this genotype correlated, directly or indirectly, with PTSD postpartum aspects (re-experience, avoidance, and hyperarousal).

**Discussion:**

Findings revealed that a lower expression of serotonin transporter gene, associated with SS genotype, seems to render women more vulnerable to depressive and PTSD symptoms after childbirth.

## Abstract 2

Supplemental Digital Content is available in the text.

## Introduction

Pregnancy has always been considered as one of the most awaited periods in a woman’s life; nevertheless, for some women, it could be combined with mild and severe disorders. Postpartum depression (PPD) is defined as an episode of major or minor depressive disorders encountered in women during the postpartum period. The Diagnostic and statistical manual of mental disorder (DSM)-IV-TR has labeled PPD ‘with onset in postpartum’ as a specific disorder that starts within 4 weeks after delivery, and in the DSM-5, it was extended to within 6 months. Literature demonstrated that PPD concerns 19.2% of women in the first 3 months, with a drop of 12.9% during the first year after birth (Gaynes *et al.*, 2005; Di Blasio *et al.*, 2015a).

Besides, recent literature has commenced talking about ‘Birth Trauma’ for women who may experience a posttraumatic stress disorder (PTSD) after childbirth. The estimated percentage of PTSD after a typical delivery ranges from 2.8 to 5.6% at 6 weeks postdelivery and 1.5% at 6 months (Ayers and Pickering, 2001), according to the diagnostic criteria of DSM-IV (Ayers and Pickering, 2001; Di Blasio *et al.*, 2015b). Percentages are more relevant, between 24 and 33%, taking count of partial posttraumatic stress symptoms (PTSS) at 4–8 weeks postpartum (Iles *et al.*, 2011).

In summary, PPD and PTSD might be counted as severe mental health problems, and for these reasons, many studies have analyzed significant risk factors behind them. Notwithstanding this, the etiology of these disorders is not adequately explained, but it is hypothesized that both environmental and biological factors may play a part. Despite several associations were highlighted with different polymorphism, most studies have focused their attention on serotonin (5-HT), a fundamental neurotransmitter in the central nervous system (David *et al.*, 2005).

Recently, many studies have reported the dysfunction of the serotoninergic system as a critical factor for the development of postpartum disorders. In this system, the serotonin transporter (5-HTT) plays a central role in the reuptake of the serotonin (Zhang *et al.*, 2015). The gene that encodes for 5-HTT is the SLC6A4, which is located on chromosome 17. Its transcriptional activity is regulated by 5-HTTLPR polymorphism characterized by an insertion/deletion of 44 base pairs in the promoter region, respectively, defined as the long (L) and the short (S) allele (Greenberg *et al.*,1996).

Given the ethnicity, it was demonstrated how the S allele or the presence of two allele L could be a risk factor for the onset of depression (Couto *et al.*, 2015). An association between PPD and S allele has been found in two studies: American research and a Chinese one (Binder *et al.*, 2010; Zhang *et al.*, 2015). Conversely, other research has revealed an association between the allele L and the insurgence of PPD (Sanjuan *et al.*, 2008; Doornbos *et al.*, 2009).

As yet, it is still an unresolved question whether the L or S allele should represent a risk factor for PPD onset. Moreover, in the last 20 years, the research has been focused on psychological aspects of birth trauma and PTSD postpartum but less on their genetic component (Ayers, 2017). There is a growing literature, however, that highlights the influence of 5-HTTLPR polymorphism on the onset of PTSD between various types of trauma such as war, natural disasters, etc. Hence, in this study, we investigated whether an association between 5-HTTLPR and postpartum depressive or PTSD symptoms could be found.

## Methods

### Study design

A longitudinal design was made with three measurements time points: time 1 (T1) at 32nd to 40th weeks gestation, time 2 (T2) at 2/3 weeks after labor, and time 3 (T3) at 3/4 months. Ethical approval was obtained from the Ethical Commission of the Catholic University of the Sacred Heart of Milan. We recruited women throughout birthing classes within the 32nd and 40th weeks of gestation in one hospital of northern Italy. Participants completed several questionnaires assessing PTSD and depression postpartum.

### Participants

The inclusion criteria to participate in the study were as follows: an adequate knowledge of the Italian language, an age 18 years upward, and Caucasian ethnicity.

One hundred fifty-five participants joined the research. One hundred forty-one women constituted the total sample at T1. At T2, the number of women active in the research was 127, and at T3, 110 women.

The frequencies of the genotypes were as follows: 36 for LL, 57 for LS, and 19 for SS.

The age of the participants was 23–42 years (*M* = 33.37, SD = 4.64), who are involved in all the three phases of the study. An estimated 81.7% had a stable occupation, and 84.5% had a medium-high level of education. Of the women participants, 77.7% were maiden, 14.6% were married, 6.8% were common-law wives, and 1% divorced. The majority of them were primipara (87.3). In total, 70.6% women had spontaneous natural childbirth without epidural analgesia, 14.7% planned cesarean, and 14.7% of women had diastolic delivery, while 11.7% had complications during pregnancy. At birth, most of the newborns were healthy: the Apgar score at minute one after delivery was 9.67 (SD = 0.89) while at 5 min was 9.93 (SD = 0.29). The dropout rate of the study is 19%.

Supplementary Table S1, Supplemental digital content 1, http://links.lww.com/PG/A257 presents the percentages of demographical variables divided by genotype. Comparing the three groups using chi-square test, we found no significant differences: age χ^2^ (2) = 2.39, *P* = 0.301; civil status χ^2^ (2) = 2.43, *P* = 0.296; education χ^2^ (2) = 3.07, *P* = 0.216; number of child χ^2^ (2) = 2.96, *P* = 0.227; type of birth χ^2^ (2) = 1.18, *P* = 0.552.

Supplementary Table S2, Supplemental digital content 1, http://links.lww.com/PG/A257 presents the descriptive statistics for PPD and PTSD among the different phases of the research.

Supplementary Tables S3, S4, and S5, Supplemental digital content 1, http://links.lww.com/PG/A257 present the descriptive statistics for PPD and PTSD divided by genotype’s groups and phases.

### Procedure

The first phase of this research (T1) was conducted during childbirth preparation courses between the 32nd and 40th week of pregnancy. Participants filled out questionnaires assessing their demographic characteristics, depression (Beck Depression Inventory II, BDI) (Beck *et al.*, 1996), and PTSD symptoms (Los Angeles Symptoms Checklist, LASC) (King *et al.*, 1995) through anonymous link of Qualtrics.

The second phase (T2) took place weeks after delivery. Questionnaires assessing childbirth’s characteristics were administered: BDI and Edinburgh Postnatal Depression Scale (EDPS) (Cox *et al.*, 1987) to evaluate depression, LASC, and PPQ (Perinatal PTSD Questionnaire) (Callahan *et al.*, 2006) to estimate PTSD. Moreover, buccal and blood samples were obtained from each woman during the recovery in the hospital.

In the third phase of the research (T3) at 3 months after childbirth, the participants were reassessed with the BDI, the EDPS, the PPQ, and the LASC in order to re-evaluate target symptoms of depression and PTSD.

### Measures

#### Depression

The BDI is a self-assessment questionnaire focusing on cognitive, motivational, affective, and behavioral symptoms of depression (Beck *et al.*, 1996). Each item is evaluated on a scale of four points (0–3), with a maximum total score of 63 provided by the total sum of items. Based on the Italian validation, a cutoff score of ≥12 evidenced the presence of depression. Scores of 13–19 are considered as mild depression; 20–28, moderate; and 29–63, severe. In this study, a good internal consistency of the instrument was identified (T1-Cronbach *α* = 0.865; T2-Cronbach *α* = 0.898; T3-Cronbach *α* = 0.597).

The EDPS is a self-report tool consisting of 10 items with a four-step Likert scale response (0–3) (Cox *et al.*, 1987). In the study, it was decided to use the cutoff of nine as advised by Benvenuti *et al.* (1999). A good internal consistency of the instrument was identified in the two different phases of the research (T2-Cronbach *α* = 0.83; T3-Cronbach *α* = 0.82).

#### Posttraumatic stress disorder

The LASC is a self-report instrument assessing the presence of PTSD symptoms, according to DSM-IV (King *et al.*, 1995). All items are rated on a five-point scale from 0 (‘not a problem’) to 4 (‘extreme problem’). The sum of the 17 items scores evaluated the PTSD gravity (Severity index), while the sum of all 43 items created a global PTSD distress index (King *et al.*, 1995). In this study, a good internal consistency of the instrument was identified in the different phases of the research (T1-Cronbach *α* = 0.793; T2-Cronbach *α* = 0.783; T3-Cronbach *α* = 0.705).

The PPQ is a measure that evaluates specifically the distress of women who are experiencing perinatal symptoms of posttraumatic distress. It is composed of 14 items ranged on a five-point scale from 0 points (not at all or never) to 4 points (very often). Scores of 19 or higher indicate significant distress (Callahan *et al.*, 2006). The cutoff highlighted possible clinical levels of PTSD symptoms are 6 or more out of 14. In this study, a good internal consistency of the instrument was identified (T2-Cronbach *α* = 0.85; T3-Cronbach *α* = 0.83).

#### Genotyping

Genomic DNA was extracted from buccal cells and peripheral blood samples, using Gentra Puregene Handbook (Qiagen, Venlo, the Netherlands), according to the manufacturer’s instructions. PCR analysis was carried out as described previously (Montirosso *et al.*, 2015), using the following primers: 5′-TCCTCCGCTTTGGCGCCTCTTCC-3′ (forward) and 5′-TGGGGGTTGCAGGGGAGATCCTG-3′ (reverse). PCR products (5-HTTLPR long allele (L) of 512 bp and short allele (S) of 469 bp) were resolved by 3% agarose gel.

### Data analysis

First, we evaluated clinical levels of PPD and PTSD in all the phases of the study. Second, the Hardy–Weinberg (H–W) was used to test the equilibrium in the sample. A test of goodness of fit was also performed to highlight any possible statistical differences between groups. Third, the assumptions of normality of the variables were verified by analyzing asymmetry, kurtosis, and normality graphs. Inferential tests, such as the Kolmogorov–Smirnov or the Shapiro–Wilk test, were also used. Preliminary analysis showed a non-normal distribution of some variables. In particular, the following variables were found out of normality: the BDI somatic scale at T2, the cognitive scale and the total at T2 and T3; the EDPS score at T3; the LASC intrusive symptoms, hyperarousal, severity and distress at T2 and the avoidance symptoms at T2 and T3; the category B symptoms of PPQ at T2. For this reason, logarithmic transformations (Log10) were used. Finally, Univariate analysis of variance (ANOVA) and moderation modeling were performed using the statistical software SPSS 20 and a macro-program PROCESS 2.1.

ANOVA analysis and moderation models had as dependent variables clinical symptoms and as independent variables or moderators, the genotype coded on three levels (LL, LS, and SS).

## Results

Descriptive statistics for PTSD and depressive symptoms are presented in the online resource (Table 1). 5-HTTLPR allelic distribution was in Hardy–Weinberg equilibrium (*P* > 0.05).

**Table 1:** Unstandardized regression coefficients and SEs for the relationship between genotype and cognitive depressive symptoms at T3 evaluated with Beck Depression Inventory

Variable	*B*	SE
Constant	1.5196***	0.3444
Cognitive symptoms	0.2203	0.1345
Genotype	0.3213	0.5632
Cognitive symptoms × genotype	0.5222*	0.2474

*P < 0.05,

**P < 0.01,

***P < 0.001.

### Outcome of analysis of variance

#### Depressive symptoms

A univariate ANOVA was carried out to verify the effect of the genotype on depressive symptoms at T1, T2, and T3. Results did not show any significant effect for the different phases (*P* > 0.05). In particular, at T1, we did not find any significant effect for the total score of BDI-II, *F* (2.109) = 0.853, *P* = 0.429, η^2^*P* = 0.015. Similarly, at T2 for the EDPS score, *F* (2.106) = 0.336, *P* = 0.716, η^2^*P* = 0.006; the BDI-II somatic scale, *F* (2.107) = 1.145, *P* = 0.322, η^2^*P* = 0.021; the BDI-II cognitive scale *F* (2.106) = 0.057, *P* = 0.945, η^2^*P* = 0.001 and the BDI-II total *F* (2.106) = 0.392, *P* = 0.677, η^2^*P* = 0.007. Also, at T3, there was not any significant effect for the EDPS score, *F* (2.103) = 2.366, *P* = 0.099, η^2^*P* = 0.044; the BDI-II somatic scale, *F* (2.95) = 0.450, *P* = 0.639, η^2^*P* = 0.009; the BDI-II cognitive scale *F* (2.103) = 0.018, *P* = 0.983, η^2^*P* = 0.000 and the BDI-II total *F* (2.103) = 0.559, *P* = 0.574, η^2^*P* = 0.011.

#### Posttraumatic symptoms

Results showed that there was a significant effect of the genotype on intrusive postpartum symptoms evaluated with LASC at T2, *F* (2.107) = 3.597, *P* = 0.03, η^2^*P* = 0.063 and with hyperarousal symptoms at T3 evaluated with PPQ, *F* (2.90) = 3.343, *P* = 0.040, η^2^*P* = 0.071. Post hoc with LSD test indicated that the mean score of SS genotype was higher at T2 (*M* = 0.506, SD = 0.322) and T3 (*M* = 4.94, SD = 3.60).

### Outcome of moderation models

#### Depressive symptoms

Interaction between cognitive symptoms at T2 and T3 was found. The overall moderation model accounted for a significant 38% of the variability in depressive scores, R2 = 0.38, *F* (5.90) = 3.19, *P* = 0.01, and the interaction for a significant 8% of the variance *F* (2.90) = 4.30, *P* = 0.016. Unstandardized regression coefficients and SEs are presented in Table 1.

Simple slopes analyses were then performed. A significant, positive relationship was identified between SS genotype and cognitive symptoms of depression at T3 (*b* = 0.7426, SE = 0.2077, *P* = 0.006).

#### Posttraumatic symptoms

First, an interaction between intrusive symptoms at T1 and T3 was discovered. Unstandardized regression coefficients and SEs are presented in Table 2.

**Table 2:** Unstandardized regression coefficients and SEs for the relationship between genotype and intrusive symptoms at T3 evaluated with Los Angeles Symptoms Checklist

Variable	*B*	SE
Constant	1.2836**	0.3444
Intrusive symptoms	0.0092	0.134
Genotype	−1.5208	0.7553
Intrusive symptoms × genotype	0.6621**	0.2088

*P < 0.05,

**P < 0.01,

***P < 0.001

The overall moderation model accounted for a significant 43% of the variability in PTSD scores, R2 = 0.43, *F* (5. 89) = 4.07, *P* = 0.002, and for 10% in the variance of the interaction *F* (2.89) = 5.97, *P* = 0.03, R2 = 0.10.

It was demonstrated that only the SS genotype predicted the intrusive symptoms at T3 (*b* = 0.6714, SEb = 0.1601, *P* = 0.001).

Second, an interaction between intrusive symptoms at T1 and T2 was identified. Unstandardized regression coefficients and SEs are presented in Table 3.

**Table 3:** Unstandardized Regression Coefficients and SEs for the Relationship Between Genotype and intrusive symptoms at T2 evaluated with Los Angeles Symptoms Checklist

Variable	*B*	SE
Constant	1.9900**	0.3811
Intrusive symptoms	0.1545	0.1996
Genotype	0.7268	0.6346
Intrusive symptoms × genotype	0.7062*	0.3233

*P < 0.05,

**P < 0.01,

***P < 0.001.

The regression result showed the significance of the general model *F* (5.98) = 4.47, *P* = 0.01, R2 = 0.18 and of the interaction explaining the 4.5% (DR2 = 0.0456) of the variance. Only the SS genotype predicted the intrusive symptoms of the postpartum (b = 0.8607, SEb = 0.2543, *P* = 0.001).

For avoidance symptoms, the first set found an interaction between symptoms at T1 and T3, *F* (5.89) = 9.84, *P* = 0.00, R2 = 0.59. The interaction was statistically significant *F* (2.89) = 11.10, *P* = 0.00, R2 = 0.16 adding 16% of the variance (DR2 = 0.016). In particular, the SS genotype was significant in predicting avoidance symptoms at 3 months.

Finally, the third set of regression evaluated the relationship between symptoms at T2 and at T3. The general model *F* (5.87) = 4.56, *P* = 0.001, R2 = 0.45 and the interaction *F* (2.87) = 3.81, *P* = 0.02, R2 = 0.06 were significant. Unstandardized regression coefficients and SEs are presented in Table 4.

**Table 4:** Unstandardized regression Coefficients and SEs for the relationship between genotype and intrusive symptoms at T3 evaluated with Los Angeles Symptoms Checklist

Variable	*B*	SE
Constant	1.5415**	0.5369
Intrusive symptoms	0.1064	0.942
Genotype	1.9223*	0.9277
Intrusive symptoms × genotype	0.6710***	0.1588

*P < 0.05,

**P < 0.01,

***P < 0.001.

In particular, the SS genotype (*b* = 0.4134, SE = 0.1243, *P* = 0.013) and the LS genotype (*b* = 0.2619, SEb = 0.1158, *P* = 0.026) were significant in predicting avoidance symptoms at 3 months.

For hyperarousal symptoms, the first set of regression evaluated the relationship between symptoms at T1 and T3. Unstandardized regression coefficients and SEs are presented in Table 5.

**Table 5:** Unstandardized regression coefficients and SEs for the relationship between genotype and avoidance symptoms at T3 evaluated with Los Angeles Symptoms Checklist

Variable	*B*	SE
Constant	1.5415**	0.5369
Avoidance symptoms	0.1064	0.942
Genotype	1.9223*	0.9277
Avoidance symptoms × genotype	0.6710***	0.1588

*P < 0.05,

**P < 0.01,

***P < 0.001.

The general model *F* (5.89) = 6.31, *P* = 0.00, R2 = 0.51 and the interaction *F* (2.89) = 3.13, *P* = 0.04, R2 = 0.05 were significant. In particular, the model showed how the SS genotype (*b* = 0.6312, SEb = 0.1509, *P* = 0.001) predicted the symptoms of hyperarousal at T3.

## Discussion

Pregnancy and the postpartum period are essential phases in women’s life delineated by biological and psychological changes. The main aim of our study was to examine the association of 5-HTTLPR polymorphism and postpartum disorders, directly and indirectly. In particular, we explored the influence of 5-HTTLPR polymorphism on depressive and PTSD symptoms immediately and at 3 months after childbirth. Compatible with previous research, we found no association between the polymorphism and the depressive symptoms (Doornbos *et al.*, 2009; Risch *et al.*, 2009; Costas *et al.*, 2010; Khabour *et al.*, 2013). Even data from multiple large samples do not seem to well support the role of candidate genes for major depression (Border et al., AJP 2019).

As other 5HTTLPR polymorphic lengths were reported in the literature (Nakamura *et al.*, 2000), it would be relevant to conduct further analyses of these sequence variants. Indeed, these variants determine the presence of possible 14 different alleles, and it would be important to determine the influence of each allele on the onset of depressive symptoms. Moreover, it would also be of interest to clarify if the S, LA, LA 5HTTLPR polymorphisms (Hu *et al.*, 2006) could represent a risk factor for PPD.

However, we demonstrated a moderation’s influence of SS genotype on cognitive symptoms evaluated with the BDI-II at T2 and T3. Preliminary data in the literature suggested that the 5-HTTLPR were correlated with the tendency of individuals to carry on negative thoughts (ruminative style) (Segal *et al.*, 2006). Individuals with SS genotype showed a higher level of rumination than individuals with LL genotype (Beevers *et al.*, 2009). Our research is the first to demonstrate a tie between polymorphism and cognitive symptoms of depression. Further investigation is needed to clarify why and how 5-HTTLPR polymorphism acts on rumination.

In this study, the analyses conducted on postnatal traumatic symptoms revealed a direct influence of SS genotype on hyperarousal symptoms at 3 months, and a significant and positive indirect effect of SS genotype on intrusive, hyperarousal, and avoidance symptoms after delivery and at 3 months. No one study has previously highlighted the association between 5-HTTLPR and PTSD for the postpartum period. This result is consistent with previous research, conducted on populations that had suffered different types of trauma (war, migration, and natural disaster). Previous studies, in fact, showed how SS genotype might trigger the appearance of intrusive thoughts, with vain attempts by subjects to suppress them (Butler *et al.*, 2003). Similarly, Liu *et al.* (2014), in a study conducted after the Wenchuan earthquake, had identified how the SS genotype predicted intrusive and avoidance symptoms, highlighting how the 5-HTTLPR polymorphism contributed to the abnormal processing of traumatic memory in PTSD. According to the dual representation theory, dreams, thoughts, and intrusive images could be recognized as aspects of traumatic memories that have not received enough elaboration (Brewin and Holmes, 2003). Taking this into account, it is likely that after childbirth, given the intense activation experienced during the delivery, some aspects of women’s narratives were encoded in a sensory and perceptual way and not in a verbalized form. Therefore, these aspects of traumatic memories re-emerged intrusively in the form of flashbacks, dreams, and vivid images, and were associated with intense negative emotions (Brewin and Holmes, 2003). According to this, it might be hypothesized that women with SS genotype experienced greater difficulty in treating the memories of traumatic birth due to hyper-activation of the amygdala, which led them to relive more frequently the event. Further, some research has also identified an association between negative thinking styles and axis hypothalamic–pituitary–adrenal. Recent discoveries suggest how negative thinking styles (ruminative, intrusive), often characterized by persevering (uncontrollable) thoughts about past or present adverse life events, prolonged cortisol secretion even after the disappearance of the stressor (Nolen-Hoeksema *et al.*, 2008). Consequently, women with SS genotype would also be stressed due to the continuous activation of intrusive thoughts and re-experience of the event.

Further, literature had also confirmed an association between SS genotype and hyperarousal symptoms in the postpartum period, as identified in our study. Grabe *et al.* (2009), for example, in their research, discovered a significant association between SS genotype and hyperarousal symptoms. A possible explanation could be, as shown above, the hyper-activation of the amygdala (Hariri *et al.*, 2002). By using noninvasive neuro-imaging sensitivity such as functional MRI, Hariri *et al.* (2002) evaluated neural activation in subjects with SS genotype during perceptual processing of facial expressions such as fear and anger. During the task, individuals with SS genotype showed almost five times more amygdala activity than subjects with LL genotype. However, the precise molecular and cellular basis of the effects of 5-HTTLPR on amygdala reactivity remains to be determined.

Further investigations are necessary for the postpartum period to better understand the association between posttraumatic symptomatology and 5-HTTLPR polymorphism. Moreover, it would be of interest to investigate also other polymorphisms that have been demonstrated to play a role in PTDS (not related to delivery). To our best knowledge, this is the first study in which a correlation between PTSD (observed during the postpartum period) and genetic polymorphism (5HTTLPR) has been found. It is known that PTSD is most significantly (genetically) correlated with major depression, but also with schizophrenia, both of which have genome-wide significant loci implicated in brain function (Gelernter *et al.*, 2019; Stein *et al.*, 2021). Therefore, advances in the understanding of the genetic mechanisms of PTSD, due to childbirth, might represent a contribution to develop novel biologically based treatment approaches for the mitigation of the disease.

### Conclusion

Our findings offer a preliminary contribution to the growing body of research on the influence of 5-HTTLPR on postpartum disorders and might have implications for the development of intervention programs to prevent women’s mental health during childbirth and the postpartum period.

## Acknowledgements

### Conflicts of interest

There are no conflicts of interest.

## Supplementary Material


