# Supplementary material for: Influence of 5-HTTLPR polymorphism on postpartum depressive and posttraumatic symptoms
Source: Psychiatr Genet. 2021 Oct 22;32(1):9–14. doi: 10.1097/YPG.0000000000000299 (PMC9904440; doi:10.1097/YPG.0000000000000299)
Supplement: Supplementary file 1 [file pg-32-9-s001.pdf]

Supplementary Table S1

Frequencies of demographical variables divided by genotype

	LL	LS	SS
Age (SD)	33.83(4,94)	32.54(4,93)	32 (3,03)
Civil status			
Maiden	77.80%	80.70%	94.40%
Married	16.70%	12.30%	5.60%
Common-law wife	2.80%	7%	
Divorced	2.70%		
Education			
Junior High	8.30%	16.10%	10.50%
Bachelor's degree	25%	30.40%	26.30%
Master's degree	58.30%	53.50%	63.20%
Other	8.40%		
Number of child			
Primipara	88.60%	85.70%	100%
>1 child	11.40%	14.30%	
Type of birth			
Spontaneous delivery	78.10%	69.80%	63.20%
C-section	9.40%	11.30%	21.10%
Dystocia	12.50%	18.90%	15.70%

Supplementary Table S2

Descriptive statistics for PPD and PTSD symptoms

	N-T1	T1		N-T2	T2		N-T3	T3	
		M	SD		M	SD		M	SD
EDPS tot				126	8.1	5.26	110	6.12	4.22
BDI somatic	141	8.09	3.84	127	7.8	5.18	110	5.54	4.07
BDI cognitive	141	1.33	2.25	127	2.13	3.35	110	1.80	2.74
BDI tot	141	9.38	5.93	127	9.88	7.94	110	7.20	5.63
LASC intrusive	141	2.79	2.096	127	1.91	2.22	107	1.49	1.47
LASC avoidance	141	4.43	4.216	126	2.98	3.54	107	2.23	2.31
LASC Hyperarousal	141	7.64	5.257	127	5.63	5.16	107	3.79	3.23
LASC Severity	141	14.87	10.13	126	10.5	9.98	108	7.47	6.09
LASC Distress	141	29.43	22.09	126	21.9	20.3	107	9.75	12.03
CatB PPQ				127	2.33	2.6	103	1.37	1.97
CatC PPQ				127	4.69	4.09	103	3.23	3.29
CatD PPQ				127	5.55	4.35	103	3.52	2.71
PPQ tot				127	12.4	9.87	103	8. 13	6.73

Supplementary Table S3

Descriptive statistics for PPD and PTSD symptoms at baseline divided by genotype

	N- T1	T1		N- T1			N- T1		
		LL			LS			SS	
		M	SD		M	SD		M	SD
EDPS tot									
BDI somatic	36	7.63	3.17	57	7.5	3.6	19	8.68	3.3
BDI cognitive	36	0.92	1.46	57	1. 04	1.75	19	1.68	2.4
BDI tot	36	8.56	4.05	57	8.54	4.51	19	10.05	5.59
LASC intrusive	36	2.61	1.91	57	2.37	1.97	19	3.11	1.99
LASC avoidance	36	3.97	3.63	57	3.77	3.69	19	4.68	3.51
LASC Hyperarousal	36	7.17	4.92	57	6.58	4.8	19	8.37	4.37
LASC Severity	36	13.75	9.29	57	12.72	8.88	19	16.16	8.75
LASC Distress	36	27.64	20.64	57	25.18	20.46	19	32.32	19.24
CatB PPQ									
CatC PPQ									
CatD PPQ									
PPQ tot									

Supplementary Table S4

Descriptive statistics for PPD and PTSD symptoms at T2 divided by genotype

	N- T2	T2		N- T2			N- T2		
		LL			LS			SS	
		M	SD		M	SD		M	SD
EDPS tot	32	7.09	4	52	8.54	6.52	19	8.11	4.45
BDI somatic	32	6.97	4.42	53	7.57	5.02	19	8.47	4.62
BDI cognitive	32	1.59	2.53	53	1.75	2.92	19	1.58	2.21
BDI tot	32	8.56	6.47	53	9.25	7.44	19	10	6.27
LASC intrusive	32	2	2.55	53	1.51	1.8	19	3.11	2.9
LASC avoidance	32	2.75	3.91	53	2.6	3.01	19	3.84	4.03
LASC Hyperarousal	32	6.19	5.39	53	5.04	4.91	19	6.95	6.4
LASC Severity	32	10.87	10.55	53	9.13	9.02	19	13.89	12.79
LASC Distress	32	20.69	18.81	53	19.83	17.15	19	29.89	29.21
CatB PPQ	32	1.53	1.86	53	2.26	2.58	19	2.84	2.71
CatC PPQ	32	4.47	3.61	53	4.38	4.03	19	4.47	4.24
CatD PPQ	32	5.28	4.01	53	4.94	4.33	19	5.58	4.68
PPQ tot	32	10.84	8.08	53	11.28	9.48	19	12.89	10.78

Supplementary Table S5

Descriptive statistics for PPD and PTSD symptoms at T3 divided by genotype

	N-T3	T3		N-T3			N-T3		
		LL			LS			SS	
		M	SD		M	SD		M	SD
EDPS tot	31	7.03	4.61	49	5.2	3.51	18	7.17	5.64
BDI somatic	31	5.1	4.6	49	5.59	4.05	18	6.28	3.92
BDI cognitive	31	1.48	1.93	49	1.59	1.96	18	1.72	2.21
BDI tot	31	7.29	4.98	49	6.12	4.3	18	8.39	6.38
LASC intrusive	29	1.31	1.31	48	1.46	1.39	18	1.89	1.77
LASC avoidance	29	2	1.64	48	1.83	1.96	18	3.33	3.49
LASC Hyperarousal	29	4.31	2.95	48	3.13	2.84	18	4.28	4.07
LASC Severity	30	7.5	4.74	48	6.42	5.14	18	9.5	8.71
LASC Distress	29	10.55	10.47	48	9.65	10.49	18	14	18.48
CatB PPQ	28	1.21	1.89	45	1.29	1.99	18	1.78	1.66
CatC PPQ	28	3.75	3.12	45	2.91	3.51	18	3.83	3.73
CatD PPQ	28	3.57	2.48	45	2.98	2.46	18	4.94	3.6
PPQ tot	28	8.54	6.26	45	7.18	6.88	18	10.56	7. 87
